# Isoniazid preventive therapy completion and factors associated with non-completion among patients on antiretroviral therapy at Kisenyi Health Centre IV, Kampala, Uganda

**DOI:** 10.1371/journal.pone.0277739

**Published:** 2023-08-22

**Authors:** Ian Amanya, Michael Muhoozi, Dickson Aruhomukama, Anthony Ssebagereka, Richard Mugambe

**Affiliations:** 1 Department of Epidemiology and Biostatistics, School of Public Health, College of Health Sciences, Makerere University, Kampala, Uganda; 2 Brainmann Analytics, Kampala, Uganda; 3 Department of Medical Microbiology, School of Biomedical Sciences, College of Health Sciences, Makerere University, Kampala, Uganda; 4 Department of Immunology and Molecular Biology, School of Biomedical Sciences, College of Health Sciences, Makerere University, Kampala, Uganda; 5 Department of Disease Control and Environmental Health, School of Public Health, College of Health Sciences, Makerere University, Kampala, Uganda; Jhpiego Nigeria, NIGERIA

## Abstract

**Background:**

Isoniazid preventive therapy (IPT) is given to HIV patients to reduce the risk of active tuberculosis (TB). However, treatment completion remains suboptimal among those that are initiated. This study aimed to determine the completion level of IPT and the factors associated with non-completion among patients on antiretroviral therapy (ART) at Kisenyi Health Center IV in Kampala, Uganda.

**Methods:**

A mixed-methods facility-based retrospective cohort study utilizing routinely collected data from 341 randomly selected HIV patients initiated on IPT was conducted. Data extracted from the registers was used to determine IPT completion. Robust Poisson regression was conducted to determine the associated factors of IPT non-completion, while in-depth interviews were conducted to explore barriers to IPT completion from the patient’s perspective.

**Results:**

A total of 341 patients who started on isoniazid (INH) were retrospectively followed up, with 69% (236/341) being female. Overall IPT completion was 83%. Multivariate analysis revealed the prevalence of IPT non-completion among males was 2.24 times the prevalence among females (aPR 2.24, 95% CI: 1.40–3.58, p = 0.001). The prevalence of IPT non-completion among patients with a non-suppressed HIV viral load was 3.00 times the prevalence among those with a suppressed HIV viral load (aPR 3.00, 95% CI: 1.44–6.65, p = 0.007). The prevalence of IPT non-completion among patients who were married, or cohabiting was 0.31 times the prevalence among those who were single (aPR 0.31, 95% CI: 0.17–0.55, p<0.000). Lack of IPT-related health education, pill burden, distance to the health facility, and patient relocation were reported as barriers to IPT completion.

**Conclusion:**

IPT completion was found to be 83% among the cohort studied. However, lower completion levels persist among males and HIV-virally non-suppressed patients. Lack of IPT-related health education, pill burden, distance to the health facility, and patient relocation were reported as barriers to IPT completion. Interventions that target these groups of people need to be intensified.

## Background

The World Health Organization (WHO) estimates a quarter of the world’s population to be infected with latent tuberculosis [1]. An estimated 10.6 million people developed TB disease in 2021, of whom 8% were co-infected with HIV [2]. In 2020, the highest numbers of TB cases were reported in the WHO regions of South-East Asia (44%), Africa (25%), and the Western Pacific (18%) [3]. In 2021, HIV-associated TB was most prevalent in Africa, and only 46% of known HIV-positive TB patients were using antiretroviral therapy (ART) [2]. Immune suppression, particularly due to HIV infection, greatly increases the risk of latent TB progressing to an active infection [4,5]. The risk of developing TB following infection is 20–37 times greater among people living with HIV (PLHIV) than among those who do not have HIV [6]. Antiretroviral therapy has been reported to reduce the risk of developing TB [7], though the risk remains higher in people with HIV, and these people are up to four times more likely to die during TB treatment [8,9].

Uganda had an estimated 1.4 million PLHIV in 2018, of whom 72% were on ART [10]. Uganda also registered about 86,000 new TB infections in 2017, of which an estimated 40% were co-infected with HIV. Among these HIV patients, TB accounted for about 30% of all deaths [1]. In Kampala, the prevalence of HIV is estimated to be about 6.9%, which is higher than the national average of 6.2% [11]. Additionally, the Uganda national TB prevalence survey of 2014–15 revealed a higher bacteriologically confirmed (B+) TB prevalence of 504/100,000 in urban areas compared to 370/100,000 in rural areas [12].

WHO recommends the use of the three I’s (isoniazid preventive therapy (IPT), intensified case finding, and infection control) to reduce the risk and burden of TB in people living with HIV [13]. IPT use reduces the risk of developing TB disease among HIV-infected individuals by about 60% [14,15], and this efficacy is even higher when accompanied by ART [16,17]. IPT prevents the progression of latent TB to active TB disease [18].

In 2014, Uganda adopted WHO guidelines that recommended the use of IPT for TB prevention as part of the comprehensive HIV/AIDS care strategy [19]. Despite the effectiveness of IPT, many countries have had little success in implementing this recommendation [1]. In Uganda, the uptake of IPT remains low, attributed to a number of factors, including: limited funding for IPT commodities; lack of awareness of the potential benefits of IPT; health facilities, districts, and implementing partners not being held accountable for IPT performance; lack of data utilization at the national, district, and health facility levels; and a lack of sustained quality mentorship, among other factors [20].

In addition, adherence and completion of treatment among patients initiated on IPT remain major concerns, as these affect its effectiveness [16]. Limited studies conducted in Ethiopia, Uganda, and Malawi, respectively, have documented completion levels of IPT ranging from 36 to 98% [21–23].

However, few studies have documented patient reasons for failure to complete the treatment in Uganda. Understanding this was key to improving the use of IPT among ART patients. Using routinely collected data, this study aimed to assess the completion of IPT and factors associated with non-completion among patients on ART. This would inform stakeholders on IPT completion, improve IPT use, and help realize its benefits, thereby reducing the risk and burden of active TB among these people.

## Objectives

### Main objective

To determine the level of IPT completion and the factors associated with non-completion among people on antiretroviral therapy at Kisenyi Health Center (HC) IV in Kampala.

### Specific objectives

1. To determine the level of IPT completion among people on antiretroviral therapy at Kisenyi Health Center IV in Kampala.
2. To determine the factors associated with IPT non-completion among patients on antiretroviral therapy at Kisenyi Health Center IV in Kampala.
3. To explore the barriers to IPT completion among ART patients from a patient’s perspective at Kisenyi Health Center IV.

## Materials and methods

### Study design

This was a mixed-methods facility-based retrospective cohort study utilizing routinely collected data. Quantitative data extracted from the facility registers was used to answer the questions on the completion of IPT and the factors associated with non-completion, while qualitative data collected through in-depth interviews with patients was used to explore the barriers to IPT completion from the patients’ perspective.

### Study setting

Kampala, Uganda’s capital, has an estimated resident population of 1.5 million people [24]. The study was conducted at Kisenyi HC IV in Kampala’s central division. Kisenyi HC IV was purposefully selected as it is the largest operational HC IV in Kampala and also receives a high volume of patients compared to smaller health facilities that provide HIV care. The facility is financed by the government but also receives funding from donors through implementing partners. Services offered by this facility are free and include TB services, HIV counseling and testing, maternity services, antenatal/elimination of mother-to-child transmission (EMTCT) services, and comprehensive ART outreach, among others. The ART and TB clinics are integrated and open from Monday through Friday to accommodate the nearly 12,000 active ART patients. To increase completion rates, IPT and antiretroviral (ARV) drug refills are given out simultaneously with synchronized durations. At the time of this study, there was an ongoing national quality improvement collaborative aimed at increasing the use of IPT among patients on ART.

### Study participants

The study participants were HIV patients on ART at Kisenyi HC IV who had been initiated on IPT. To reduce recall bias, a total of 1166 ART patients who had been initiated on IPT in September 2019 at Kisenyi HC IV were considered.

### Inclusion criteria for the quantitative component

Only ART patients initiated on IPT in September 2019 were included in the study.

Purposively selected non-completing ART patients for in-depth interviews who provided informed consent were also included in the study.

### Exclusion criteria for the quantitative component

ART patients initiated on IPT in any period other than September 2019 were excluded from the study, as were those whose records could not be traced or found at the facility.

### Exclusion criteria for the qualitative component

Patients who were ill and unavailable at the time of data collection and those who had no phone contact were excluded from in-depth interviews.

### Study variables

#### Dependent variable

IPT non-completion, which was measured as a person failing to pick up their fifth consecutive monthly refill within 6 months of initiation, was the dependent variable. On the other hand, IPT completion was defined as a patient picking up their fifth INH refill within 6 months of initiation.

Patients who had begun IPT had to return to the health facility every month for five months, starting after a 2-week assessment for any self-reported adverse effects. IPT refills were given by clinicians at the monthly visit, and they also evaluated the patient’s adherence by pill count. INH was prescribed for multiple months to patients who received ART on a multi-month dispensation. Data on completion was extracted from the IPT registers, patient ART cards, and Uganda electronic medical records (UgandaEMR) system.

#### Independent variables

The independent variables included age, sex, weight, height, body mass index (BMI), marital status, WHO HIV clinical stage at IPT initiation, and pre-IPT HIV viral suppression (defined as the most recent measurement within 12 months of IPT initiation). A patient was considered HIV-virally suppressed if they had a result of fewer than 1000 viral copies per milliliter of blood [25]. Data on independent variables were extracted from the IPT registers, patient ART cards, and UgandaEMR.

#### Data sources and measurement

Data collection ran for one month between November and December 2020. Quantitative data were extracted from the IPT register, ART cards, and the Uganda Electronic Medical Records System (EMR) using a structured data tool. Variables captured quantitatively were residence (district, sub-county, village), WHO HIV stage at the time of IPT initiation, IPT refill and next appointment dates, weight (in kilograms), height (in centimeters), age (completed years), sex (male or female), body mass index (BMI, derived from height and weight), marital status, and pre-IPT viral load result. In-depth interviews that lasted 20–30 minutes were conducted by a trained research assistant with the help of an interview guide. Variables explored qualitatively included IPT-related health education, challenges encountered while on IPT, and smoking and/or alcohol consumption. These variables gave rise to some of the themes presented in the findings. All qualitative interviews were recorded with a call recording application and notes taken during the interviews. Interviews were conducted until saturation was reached.

#### Bias

Potential bias at the participant selection stage was addressed by employing simple random sampling. The selection of respondents for in-depth interviews was also stratified. Recall bias among in-depth interview (IDI) respondents was minimized as the interviews were conducted within 9 months after the expected completion date.

### Sample size considerations

To determine the completion of IPT and the factors associated with non-completion, the Yamane formula at a 0.05 level of precision was used [26]. This formula was used due to the finite population of patients initiating IPT in a selected month.

$$\text{n}=\text{N}/\left[1+\text{N}{\left(\text{E}\right)}^{2}\right]$$

Where, n = sample size, N = population size, E = level of precision.

n = 1166/ [1+1166 (0.05) ^2^]

n = 298

Adjusting for a 10% loss to follow-up

The minimum sample size required was 331.

### Sampling procedure

**Fig 1:**
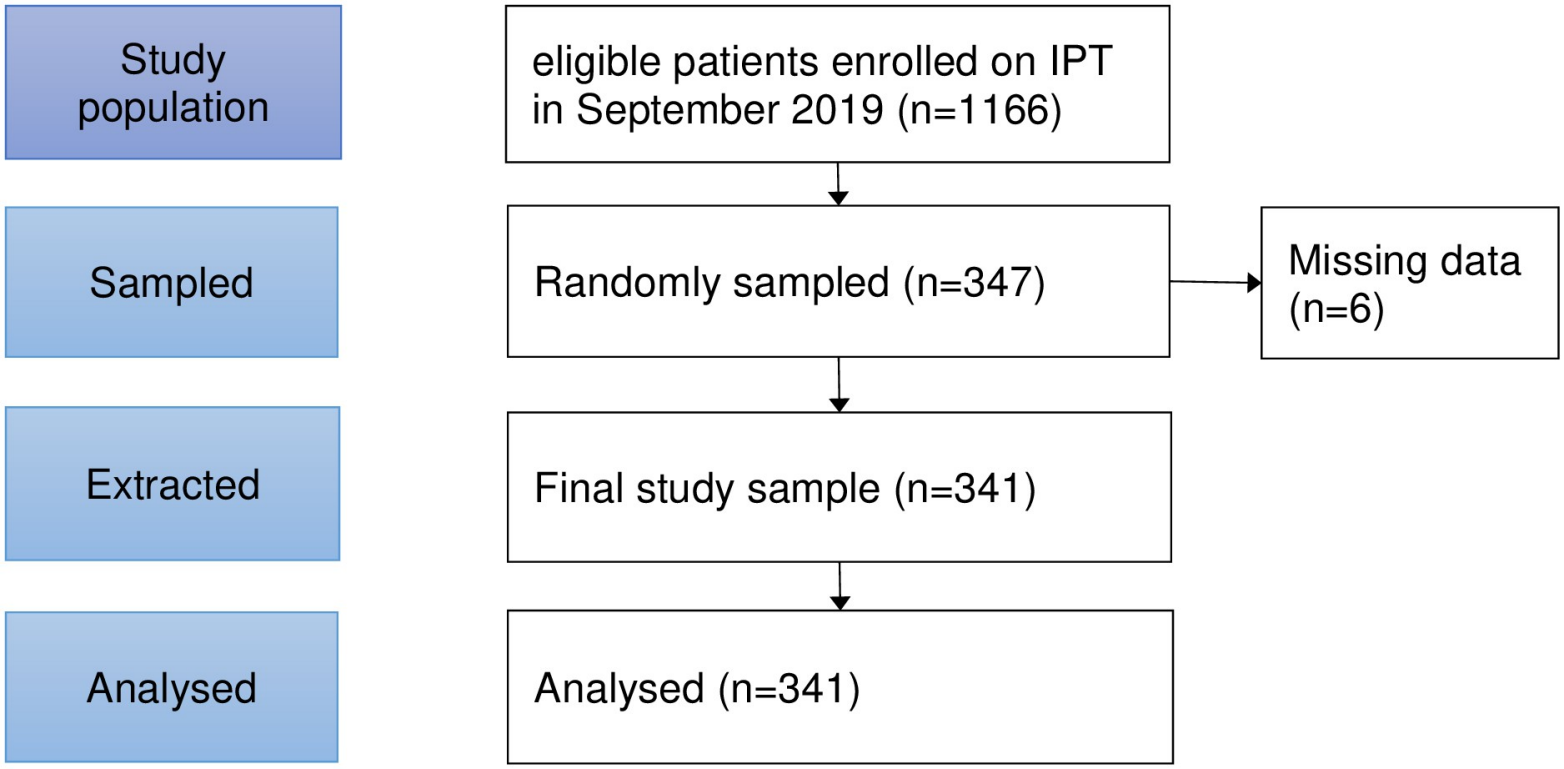
Flow chart illustrating participant recruitment in the study.

A list of IDs of patients initiated on IPT during September 2019 was compiled. Simple random sampling was used to select the 347 patients using Microsoft Excel. Their records were then extracted from the IPT registers into the data abstraction tool. Fig 1 below illustrates how the participants were selected for inclusion in the study. Respondents for in-depth interviews were purposefully selected from the list of non-completers. The respondent selection was stratified by age group, sex, marital status, and district of residence.

### Data analysis

#### Quantitative data analysis

The entry, cleaning, and sorting of data were all parts of the data management process. The data was entered into a specially designed Excel data screen. The data was then thoroughly examined for completeness and logical flow. Participants with missing variables were excluded from the data analysis.

The characteristics of the study participants were described through descriptive analysis. Additionally, among those who started IPT, the percentage of participants who got their fifth consecutive monthly refill within 6 months was determined.

Simple Poisson regressions of the outcome and the predictor were run to determine factors associated with non-completion to be included in the multivariate model. Variables with a p-value less than 0.2 were considered statistically significant for inclusion in multivariate analysis.

A robust Poisson regression model was run to determine the predictors of IPT non-completion. The study had a binary outcome but used a robust Poisson model instead of logistic regression, which would have been suitable if the prevalence of IPT non-completion was under 10%. The use of logistic regression that gives odds ratios tends to overestimate the strength of association in such scenarios [27]. Variables were checked for multicollinearity before being added to the model. Variables with a p-value less than 0.05 were significantly associated with IPT non-completion.

#### Qualitative data analysis

All audio recordings were simultaneously transcribed and translated into English verbatim. Transcripts were then proofread for accuracy by the principal investigator (PI) before being uploaded to Atlas.ti Version 6.0 for analysis. Both inductive and deductive coding approaches were applied to generate the codebook [28,29]. The codebook generated during analysis was reviewed by the PI before adoption. Following a review, some codes were combined; others were dropped or renamed to fit under emerging themes. The results were presented as narratives along with supporting quotes per theme. The analysis was regularly reviewed by the PI at every stage to ensure the reflexivity of the interpretations.

### Ethical considerations

The research assistants were trained on how to ensure privacy and confidentiality for all participants both during and after data collection. Data collection took place at the height of the COVID-19 pandemic. Consequentially, in-depth interviews were conducted over the phone in place of physical interviews, and recorded consent was also obtained from the respondents for infection prevention and control (IPC) reasons. The respondent was asked to confirm their clinic number. The interview was then assigned a unique identifier for the proceeding transcription and analysis processes. Ethical approval for this study was sought and granted before the study by the Makerere University School of Public Health Higher Degrees, Research, and Ethics Committee. Permissions were subsequently granted by the director of public health environment at Kampala Capital City Authority (KCCA) and the in-charge Kisenyi HC IV.

## Results

### Description of the study population

A total of 341 records of participants were included in the analysis. About (31%, 104/341) of the participants were aged 29 years and below. The median age was 33 (IQR 28–40). Most of the respondents were female (69%, 236/341) and (54.5%, 186) had a normal body weight. Additionally, (97.1%, 331/341) of the participants were at WHO HIV clinical stage 1. All the participants had a viral load test done in the last 12 months, with (97%, 330) having a suppressed result. Regarding marital status, (58.4%, 199/341) of the participants were married or cohabiting while (20.5%, 70) were divorced or separated (Table 1).

**Table 1:** Characteristics of the study population.

Characteristic	Frequency		
Age	Non-Completers % (n) 17% (58)	Completers % (n) 83% (283)	Overall (n)
**0–29**	20.2% (21)	79.8% (83)	104
**30+**	15.6% (37)	84.4% (200)	237
**Sex**			
**Female**	13.6% (32)	86.4% (204)	236
**Male**	24.8% (26)	75.2% (79)	105
**BMI**			
**Normal weight**	19.9% (37)	80.1% (149)	186
**Underweight**	22.2% (4)	77.8% (14)	18
**Pre-obesity**	14.1% (12)	85.9% (73)	85
**Class 1 obesity**	12.1% (4)	87.9% (29)	33
**Class 2&3 obesity**	5.3% (1)	94.7% (18)	19
**WHO HIV clinical stage**			
**Stage 1**	16.9% (56)	83.1% (275)	331
**Stage 2&3**	20.0% (2)	80.0% (8)	10
**HIV Viral suppression**			
**Suppressed**	15.8% (52)	84.2% (278)	330
**Non-suppressed**	54.6% (6)	45.4% (5)	11
**Marital status**			
**Single**	31.7% (19)	68.3% (41)	60
**Married/cohabiting**	9.5% (19)	90.5% (180)	199
**Divorced/separated**	24.3% (17)	75.7% (53)	70
**widowed**	25% (3)	75% (9)	12

### IPT completion

Of the 341 patients who started INH, (83%, 283) completed the dose, compared to (17%, 58) who did not. Among female patients, (86.4%, 204) of those who initiated IPT completed it, whereas (13.6%, 32) did not. Among males, (75.2%, 79) completed IPT, while (24.8%, 26) did not. Among patients with a suppressed HIV viral load, (84.2%, 278) completed IPT while (15.8%, 52) did not. Among patients with a non-suppressed HIV viral load, (54.6%, 6) did not complete IPT, compared to (45.4%, 5) who did. IPT was successfully completed by (79.8%, 83) of patients who were under the age of 30, compared to (20.2%, 21) who did not. About (91%, 180) of the patients who were married or cohabitated completed IPT, compared to (9.5%, 19) who did not.

### Factors associated with IPT non-completion

Factors associated with IPT non-completion, both at bivariate and multivariate analysis are presented in Table 2 below.

**Table 2:** Bivariate and multivariate analysis.

Variable	Completed IPT No % (n) Yes % (n)		Crude Prevalence Ratios	Adjusted Prevalence Ratios (aPR)
**Overall**	**17% (58)**	**83% (283)**	**N/A**	**N/A**
**Age**				
0–29	20.2% (21)	79.8% (83)	1.00	—
30+	15.6% (37)	84.4% (200)	0.77 (0.48–1.25)	—
**Sex**				
Female	13.6% (32)	86.4% (204)	1.00	1.00
Male	24.8% (26)	75.2% (79)	1.83 (1.15–2.91) *	2.24 (1.40–3.58) **
**WHO HIV clinical stage**				
Stage 1	16.9% (56)	83.1% (275)	1.00	—
Stage 2&3	20.0% (2)	80.0% (8)	1.18 (0.33–4.19)	—
**HIV Viral load status**				
Suppressed	15.8 (52)	84.2 (278)	1.00	1.00
Non-suppressed	54.6 (6)	45.4 (5)	3.46 (1.91–6.28) ***	3.00 (1.35–6.65) **
**Marital status**				
Single	31.7 (19)	68.3 (41)	1.00	1.00
Divorced/separated	24.3 (17)	75.7 (53)	0.77 (0.44–1.34)	0.77 (0.43–1.38)
Married/cohabiting	9.5 (19)	90.5 (180)	0.30 (0.17–0.53) ***	0.31(0.17–0.55) ***
Widowed	25 (3)	75 (9)	0.79 (0.28–2.26)	1.07 (0.35–3.24)
**BMI**				
Normal weight	19.9% (37)	80.1% (149)	1.00	—
Underweight	22.2% (4)	77.8% (14)	1.12 (0.45–2.78)	—
Pre-obesity	14.1% (12)	85.9% (73)	0.71 (0.40–1.30)	—
Class 1 obesity	12.1% (4)	87.9% (29)	0.61 (0.23–1.60)	—
Class 2&3 obesity	5.3% (1)	94.7% (18)	0.26 (0.38–1.83)	—

Level of significance p<0.05*; <0.01**; <0.001***.

### Bivariate analysis

The prevalence of IPT non-completion among patients aged 30 years was 0.77 times the prevalence among those aged 29 years and below (Crude PR 0.77, CI: 0.48–1.25). The prevalence of IPT non-completion among males was 1.83 times the prevalence among females (Crude PR 1.83, 95% CI: 1.15–2.91). The prevalence of IPT non-completion among patients with a non-suppressed HIV viral load was 3.46 times the prevalence among those with a suppressed HIV viral load (Crude PR 3.46, 95% CI: 1.91–6.28). The prevalence of IPT non-completion among patients who were married, or cohabiting was 0.30 times the prevalence among those who were single (Crude PR 0.30, 95% CI: 0.17–0.53).

### Multivariate analysis

In multivariate analysis, factors significantly associated with IPT non-completion were sex, HIV viral suppression, and marital status. The prevalence of IPT non-completion among males was 2.24 times the prevalence among females (aPR 2.24, 95% CI: 1.40–3.58, p = 0.001). The prevalence of IPT non-completion among patients with a non-suppressed HIV viral load was 3.00 times the prevalence among those with a suppressed HIV viral load (aPR 3.00, 95% CI: 1.35–6.65, p = 0.007). The prevalence of IPT non-completion among patients who were married, or cohabiting was 0.31 times the prevalence among those who were single (aPR 0.31, 95% CI: 0.17–0.55, p<0.000).

### Barriers to IPT completion

The themes and subthemes that emerged were.

**Theme**: knowledge about IPT.

**Theme**: Challenges encountered while taking IPT. **Subthemes**: lack of transport, distance to the health facility, pill burden, and relocation.

### Theme; Knowledge about IPT

One of the reasons for noncompliance was the absence of IPT-related health education. The respondent was unaware of his IPT initiation.

*“…I met a friend at the garage where I work who disclosed that he was on drugs and had not received IPT all his life*. *So*, *I wondered why I had been given these tablets*, *yet the doctor didn’t tell me I had TB*, *I swallowed them for 2 months and got tired and stopped*”

single male patient.

### Theme: Challenges encountered while taking IPT

#### Subtheme: High pill burden

A high pill burden was another reason for IPT non-completion. This was more pronounced among patients who had to take other drugs for other pre-existing conditions, such as hypertension, in addition to ART and IPT.

“…*the tablets to swallow are very many*, *I have these ART tablets*, *then my hypertension tablets*, *and then the IPT*. *So*, *at times I’d forget to swallow the IPT as it wasn’t treating me for anything*, *and I completely stopped after forgetting it thrice*”

Female Patient.

#### Subtheme: Distance to the health facility

The distance to the health facility was cited as a challenge, as some of the patients could not afford transport fares every month to the health facility to pick up their IPT doses.

“…*the first challenge*, *most of us come from far and at the beginning they first gave me for a month and most of my work I am casually employed*. *So sometimes it would be hard for me to tell my boss there is somewhere I am going I will not be around now*”

Male Patient.

#### Subtheme: Relocation

A change of business and relocation from one place to another were cited as other reasons for IPT non-completion.

“…*when I shifted from Kisenyi to Kitebi*, *I didn’t go with my card and when I went to get my drugs from Kitebi*, *they didn’t give me IPT yet in Kisenyi they had given me for three months*. *I explained to the doctors*, *but they told me they’d give me next time until I got fed up and returned to Kisenyi where they have again started me on IPT again*”

Female patient.

## Discussion

### IPT completion

The goal of the study was to ascertain the IPT completion rate and related variables among those receiving antiretroviral therapy at Kisenyi Health Center IV in Kampala. The cohort of patients under study had an IPT completion rate of 83%, which was a comparatively high rate. This result is comparable to research from Zimbabwe and India, where studies [30,31] indicated that 81% of HIV patients have completed IPT. This completion rate is higher than that of the SEARCH HIV test and treat trial, which was done in five communities in Uganda [32] and which Tram et al. reported at 73%.

The higher completion level found by our study can be attributed to the efforts of the national Quality Improvement (QI) collaborative, which was founded in January 2019 to improve IPT completion as part of a wider continuous quality improvement effort in Uganda. All 14 of Uganda’s health regions, 126 of its 127 districts, and 739 ART-accredited sites were included in the collaborative. A situational analysis of IPT completion at high-volume health facilities was done as part of the QI collaborative, and these facilities received coaching support. Regional coaches worked with district-based coaches to help facility teams identify the causes of poor IPT completion, identify interventions, and create action plans to improve IPT completion. As a result of coaching help, facilities were able to develop local strategies to increase IPT completion while also testing approaches from other sites [33].

Most patients receiving INH concurrently with ART lowers the expense of obtaining medication because they would not need to make as many trips to the medical institution, which contributed to the relatively high completion rate. Even though this study indicated that IPT completion rates have increased over time, 17% of patients who began IPT did not finish the treatment course. If IPT is not completed, its effectiveness is reduced, and the patient is once again in danger of developing active TB, which raises morbidity and mortality.

### Factors associated with IPT non-completion among patients on ART

Males were shown to have a higher prevalence of IPT non-completion than females. This finding is consistent with a study conducted in rural Malawi by Little et al., which found that males were more likely than females to fail to complete the study [34]; the typical masculine conduct, lower uptake of facility-based services, cultural precepts, and societal conventions that obstruct men’s health-seeking activities and lead to subpar treatment outcomes can all be used to explain this. To increase completion rates, focused follow-up with male patients undergoing IPT is necessary.

The study also found that patients who had a non-suppressed HIV viral load had a higher prevalence of IPT non-completion. HIV viral non-suppression has been reported to be associated with, among other factors, adherence, defined as a patient’s ability to follow a treatment plan, take medications at prescribed times and frequencies, and follow restrictions regarding food and other medications [35]. Patients who are HIV-virally non-suppressed are more likely to have poor ART adherence [36,37]. This, therefore, explains the association between IPT non-completion and viral non-suppression among HIV patients. Patients who did not complete IPT were more likely to be HIV-virally non-suppressed. It is possible that HIV-virally non-suppressed patients were likely to be non-adherent to ART and other associated medicines, including IPT. Targeted follow-up of HIV-virally non-suppressing patients on IPT is needed to reduce the prevalence of IPT non-completion in this group.

Additionally, the study found that marriage was strongly associated with a lower rate of IPT non-completion than being single. Married individuals are more likely to be open about their status with their partners [38], which offers emotional and psychological support [39], which has been linked to improved treatment outcomes [40,41].

This can also be explained by the fact that marriage gives patients stability by allowing them to be taken care of while undergoing therapy. IPT completion rates are expected to increase if these patients’ wives provide them meals on time and remind them to take their medications. This finding highlights the importance of providing ART patients, as well as those on IPT, with social support networks. These patients can benefit from social and psychological support from services like psychosocial and adherence counseling as part of the HIV care program [42].

### Barriers to IPT completion

Patient-related barriers to IPT completion were explored in this study.

#### Lack of health education

To improve health-related patient literacy, knowledge and life skills that support individual and communal health need to be shared with patients. Numerous studies have shown that health education can enhance the effectiveness of patient care [43,44]. On the other hand, the lack of health education among HIV patients is related to lower patient adherence and poor treatment outcomes [45,46]. Due to the large volumes of patients that the health workers attend to on clinic days [25,47], they tend to focus on providing drug refills while neglecting to provide health education [48]. In these circumstances, patients receive drug refills without health education, resulting in non-completion.

#### High pill burden

Some of the respondents abandoned IPT due to the pill burden. This finding is similar to studies conducted in Kenya, Swaziland, Zimbabwe, and India, which found that pill burden was one of the reasons for IPT non-completion, especially for patients who had to take other drugs for underlying conditions in addition to ART [31,49–52]. Concurrent management of multiple conditions can be difficult due to drug-drug interactions [53]. IPT regimens with shorter durations and less frequent dosages, like 3-HP, 4R, and 3-4HR, should be considered for patients with pill burden challenges to improve IPT completion levels.

#### Distance to the health facility

This was also cited as one of the barriers to IPT completion, as patients had difficulty accessing IPT doses due to a lack of transportation. This echoes findings from studies conducted in Tanzania, Zimbabwe, and Ethiopia, which found that patients were discouraged by the need to attend clinic every month combined with the long treatment duration of IPT [30,54,55]. A significant proportion of patients do not get ART from their nearest facility [56,57] due to various reasons that include the fear of stigma and the perception that care at local facilities is of lower quality compared to that at distant ones [57,58]. Uganda adopted differentiated service delivery (DSD) models in 2016 for both HIV testing services and treatment to cater to the needs and preferences of the increasing number of clients as well as improve the quality of services and efficiency in service delivery [25]. These DSD models can be further utilized for giving IPT refills to patients in addition to ART.

#### Relocation

When patients relocate to new areas within or outside of Kampala, they also transfer to new, nearby health facilities. Most of these patients transfer to these sites without proper referral documentation, in what is termed “self-transfer” [59]. In this case, the transfer-in facility may not offer care continuity due to a lack of relevant referral documentation [60]. This was also echoed in a study done in Dar es Salaam, where people living with HIV who were transferred to other clinics within the Dar es Salaam region after IPT initiation had significantly lower IPT completion levels compared to those that attended the same clinic [55]. These findings are also similar to those of studies conducted in Zimbabwe and Ethiopia [30,61]. Linking patient databases to improve access and continuity of care can be explored to improve not only IPT completion but also other treatments like ART.

### Study limitations

The study’s limitations were mainly related to missing and incomplete data since this was a retrospective record review of routinely collected program data. This was overcome by triangulating data from different sources, i.e., the IPT, ART registers, ART cards, and the UgandaEMR.

The study was conducted in one health facility in Kampala city, and hence the findings may not be generalizable to other health facilities within other cities and districts in Uganda. Treatment completion was assessed by a patient picking up a refill as a proxy for actual consumption of isoniazid. It is, however, possible that patients might have returned to pick up another refill without having consumed all the tablets from the previous refill.

The study also explored barriers to IPT completion from the patient’s perspective and did not look at provider- or system-related barriers to IPT completion. To effectively improve IPT use, barriers to its completion from the provider’s perspective also need to be explored.

Some of the planned interviews by stratification were not conducted as there were no patients or respondents in those strata. The barriers to IPT completion presented represent the patient categories that were interviewed.

The barriers to IPT completion explored may have been limited by social desirability bias. This was mitigated by employing unconditional positive regard [62].

### Recommendations

1. Health caregivers or health workers need to provide patients with IPT-related health education before IPT initiation. Opportunities to give group health education on clinic days can be leveraged. Patient attitudes towards the therapy and, consequently, completion levels are likely to improve when patients are fully aware of why they are being initiated on the treatment.
2. There’s a need for the Ministry of Health and partners to increase availability and access to other regimens of IPT with less frequent dosages compared to a 6- to 9-month daily dose of INH. This will reduce the pill burden, especially for patients with comorbidities, and improve adherence and completion rates.
3. Health workers and health caregivers should provide and intensify treatment adherence support and counseling to HIV patients who are virally non-suppressed, not only for ART but also for IPT.
4. There’s a need for the Ministry of Health to explore the use of biometric systems and link ART or TB clinics across health facilities to enable patients, especially the mobile populations, to access ART and IPT at any accredited health facility with accurate and timely documentation.
5. Health facilities should take advantage of community DSD models and exploit them to deliver IPT in addition to ART and other related medications to eligible patients. Additionally, multi-month ART refills synchronized with IPT should also be taken advantage of to improve uptake and completion of IPT.
6. There’s a need for further research on factors influencing IPT completion from the health system’s and provider’s perspectives.

## Conclusion

The IPT completion level was found to be relatively high, at 83% among the cohort of patients, but still less than the expected 95%. This corresponds with a general improvement in HIV services and the monitoring of HIV services by health facilities and stakeholders. Being male and being HIV-virally unsuppressed were significantly associated with a higher prevalence of IPT non-completion. Being married was associated with a lower prevalence of IPT non-completion. Lack of health education, pill burden, distance to the health facility, and relocation were reported as the reasons for failure to complete IPT.
